# Differential DNA methylation associated with delayed cerebral ischemia after aneurysmal subarachnoid hemorrhage: a systematic review

**DOI:** 10.1007/s10143-024-02381-5

**Published:** 2024-04-10

**Authors:** Tomasz Klepinowski, Bartłomiej Pala, Samuel D. Pettersson, Kajetan Łątka, Dominik Taterra, Christopher S. Ogilvy, Leszek Sagan

**Affiliations:** 1 Department of Neurosurgery, Pomeranian Medical University Hospital No. 1, Szczecin, Poland; 2 Neurosurgical Service, Beth Israel Deaconess Medical Center, Harvard Medical School, Boston, MA USA; 3 Department of Neurology, St Hedwig’s Regional Specialist Hospital, Institute of Medical Sciences, University of Opole, Opole, Poland; 4 Department of Orthopedics and Rehabilitation, Jagiellonian University Medical College, Zakopane, Poland

**Keywords:** Delayed cerebral ischemia, Cerebral vasospasm, Aneurysmal subarachnoid hemorrhage, DNA methylation, Epigenetic changes, Intracranial aneurysm

## Abstract

Recent studies suggest that differential DNA methylation could play a role in the mechanism of cerebral vasospasm (CVS) and delayed cerebral ischemia (DCI) after aneurysmal subarachnoid hemorrhage (aSAH). Considering the significance of this matter and a lack of effective prophylaxis against DCI, we aim to summarize the current state of knowledge regarding their associations with DNA methylation and identify the gaps for a future trial. PubMed MEDLINE, Scopus, and Web of Science were searched by two authors in three waves for relevant DNA methylation association studies in DCI after aSAH. PRISMA checklist was followed for a systematic structure. STROBE statement was used to assess the quality and risk of bias within studies. This research was funded by the National Science Centre, Poland (grant number 2021/41/N/NZ2/00844). Of 70 records, 7 peer-reviewed articles met the eligibility criteria. Five studies used a candidate gene approach, three were epigenome-wide association studies (EWAS), one utilized bioinformatics of the previous EWAS, with two studies using more than one approach. Methylation status of four cytosine-guanine dinucleotides (CpGs) related to four distinct genes (ITPR3, HAMP, INSR, CDHR5) have been found significantly or suggestively associated with DCI after aSAH. Analysis of epigenetic clocks yielded significant association of lower age acceleration with radiological CVS but not with DCI. Hub genes for hypermethylation (VHL, KIF3A, KIFAP3, RACGAP1, OPRM1) and hypomethylation (ALB, IL5) in DCI have been indicated through bioinformatics analysis. As none of the CpGs overlapped across the studies, meta-analysis was not applicable. The identified methylation sites might potentially serve as a biomarker for early diagnosis of DCI after aSAH in future. However, a lack of overlapping results prompts the need for large-scale multicenter studies. Challenges and prospects are discussed.

**Supplementary Information:**

The online version contains supplementary material available at 10.1007/s10143-024-02381-5.

## Introduction

Intracranial aneurysms (IAs) can be appreciated in neuroimaging of approximately 3% of population [1]. Although global trends for treatment of unruptured IAs have shifted in terms of dome size and age of the treated patients [2, 3], their annual rupture rate remains on average 9 per 100,000 population and is still related to high mortality and morbidity [4]. Survivors are at risk of various complications, one of which is cerebral vasospasm (CVS) and delayed cerebral ischemia (DCI). The latter one is a clinical presentation of CVS and has been defined as a new focal deficit (hemiparesis, dysphasia) or decrease in Glasgow Coma Scale by two points not attributable to hydrocephalus, rebleeding, seizures, or hyponatremia.

Despite the importance, basics of the CVS have not been fully fathomed. Directly, it is caused by contraction of the arterial smooth muscles due to insufficiency of the vasodilators, excessive activity of vasoconstrictors, or most likely both. Also, components of the extravasated blood could play a significant role. In vitro, once pure oxyhaemoglobin contacts albumins of the arterial surface, the vascular contraction follows. However, tests with superoxide dismutase and catalase failed to prevent this vasospasm [5]. Although haemoglobin deletes a vasorelaxant nitric oxide, numerous agents aiming to relax the vessels (sodium nitroprusside, endothelin-1 antagonists, nicardipine, diltiazem) failed to fully prevent or reverse the vasospasm [5–7]. Furthermore, both inflammation and immunoreactivity have been implicated in the vasospasm, yet immunomodulators do not reduce its prevalence [5]. Although proliferative vasculopathy was noticed in some vessels after subarachnoid hemorrhage, it does not explain its abrupt radiographic and clinical onset as well as its transient nature. Finally, since 2019 epigenetics in the predisposition to or role in the mechanism of vasospasm were started to be appreciated.

Epigenetics describes alterations in gene expression that could be heritable and that do not entail change in DNA sequence. These alterations, such as DNA methylation, histone modification, or non-coding RNA are affected by many environmental factors as well as by lifestyle. Most attention has been given to DNA methylation as it is, according to the current knowledge, the only epigenetic mechanism able to induce inheritable changes in the gene expression that could be transmitted through the germline [8]. DNA methylation commonly occurs in the regions of cytosine-guanine dinucleotides (CpGs). If CpGs are clustered over 200 base pairs and encompass percentage greater than 50%, then they are termed CpG islands [9]. Hypermethylation CpG sites serve as regulatory mechanism of gene expression by destabilization of nucleosomes, recruitment of the related proteins, remodeling of the chromatin structure and ultimately inhibition of transcription [9]. Availability of the epigenome-wide DNA methylation profiling with high-throughput arrays opens up vast horizons of possibilities since it is cost-effective, highly sensitive, and does not require large amount of input genomic DNA (gDNA). Such differentially methylated CpGs sites identified through epigenome-wide association studies (EWAS) are then often verified in replication studies with a gene candidate approach.

To date, there has been no systematic review regarding the role of epigenetics in the CVS and DCI after the aneurysmal subarachnoid hemorrhage (aSAH). Considering the global and regional socioeconomic burden of the aSAH with DCI and lack of effective prophylaxis or treatment, it is of great importance to seek novel diagnostic biomarkers and potential therapeutic targets. This study aims to provide an answer to the following PICO (Population – Intervention – Comparison – Outcome) framework question: are there differentially methylated DNA sites in blood or cerebrospinal fluid of adult subjects with DCI after aSAH, as compared with those without DCI after aSAH?

## Methods

### Study design and search strategy

This is a systematic review of DNA methylation association studies involving participants with aneurysmal subarachnoid hemorrhage complicated by clinically relevant vasospasm termed as delayed cerebral ischemia. For transparency and good research practice, Preferred Reporting Items for Systematic Reviews and Meta-Analyses (PRISMA) 2020 guidelines were adopted (Supplementary Material 2). No temporal or language restrictions were imposed. Due to the recent advances in epigenetic analysis, the search was conducted in three waves: the first wave in June 2021, the second wave in June 2022, and the last complementary wave on 3 February 2024 just prior to submission to ensure up-to-dateness. PubMed MEDLINE, Scopus, and Web of Science databases were searched by two authors (T.K. and B.P.). Additional articles were sought through the references. Titles were imported into EndNote 21.2, which was also utilized for bibliography. The full electronic search strategy can be viewed as Supplementary Material 1.

### Eligibility

We included only original peer-reviewed DNA methylation association studies in humans that reported link between DNA methylation changes (either epigenome-wide or region-specific) and delayed cerebral ischemia after subarachnoid hemorrhage from the ruptured intracranial aneurysm. The exclusion criteria were as follows: (1) extracranial aneurysms, (2) letters to editors, (3) reviews, and (4) short responses, (5) animal studies. Eligibility was assessed by T.K. and B.P. In case of disagreement, the senior researcher (L.S.) was reached for consensus. Next, data extraction was performed.

### Data extraction and curation

The articles that were deemed eligible were read thoroughly so as to extract and curate the following data: (1) mean time from aSAH to sample collection, (2) biological specimen used for DNA methylation profiling, (3) scope of DNA methylation assessment (epigenome-wide versus region-specific targeting or candidate gene approach), (4) geographical region and ethnicity, (5) DCI definition, (6) epigenetic mechanism (7) number of subjects, (8) age, (9) sex, (10) number of CpGs tested, (11) CpG annotations, (12) DNA methylation details, and (13) clinical significance of the methylation alterations. To extract data regarding CpG sites, Infinium MethylationEPIC v1.0 B4 Manifest file was uploaded to RStudio version 2023.06.0 Build 421and read in tabular display. Genes associated with or nearest to the CpG sites were read either from UCSC_RefGene_Name or GencodeCompV12_NAME columns (in this order). Other details extracted from this manifest included chromosome number, relation to CpG island, and region of the gene associated with a given CpG site. In cases of no results yielded by search of the manifest, genome browser of University of California, Santa Cruz was consulted (https://genome.ucsc.edu/). The extracted data were curated in a spreadsheet of Excel Microsoft 365 version 2401 (Redmond, USA)—see Supplementary Material 1.

### Risk of bias and quality assessment

To assess quality and risk of bias, studies included in this systematic review were evaluated using Strengthening the Reporting of Observational Studies in Epidemiology (STROBE) checklist. Each of the 22 items of this statement was attributed a point for the positive answer (see Supplementary Material 3). As described by us elsewhere [10], if the number of points was below 11 (50% of the maximum STROBE score), then such an article was deemed to be of low quality. If a study addressed between 11 and 14 items, moderate quality was assigned. In case of 15–18 or more than 18 points, then high or very high quality was accepted, respectively.

## Results

**Fig. 1:**
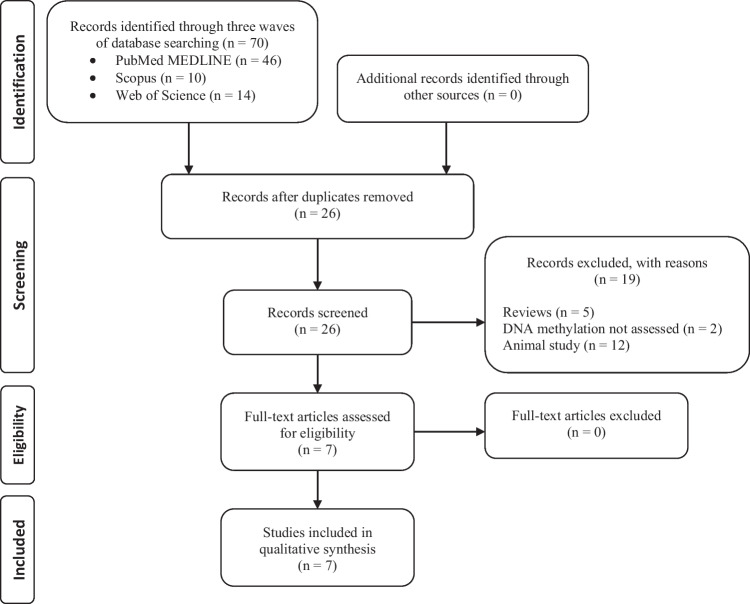
Study selection process

**Fig. 2:**
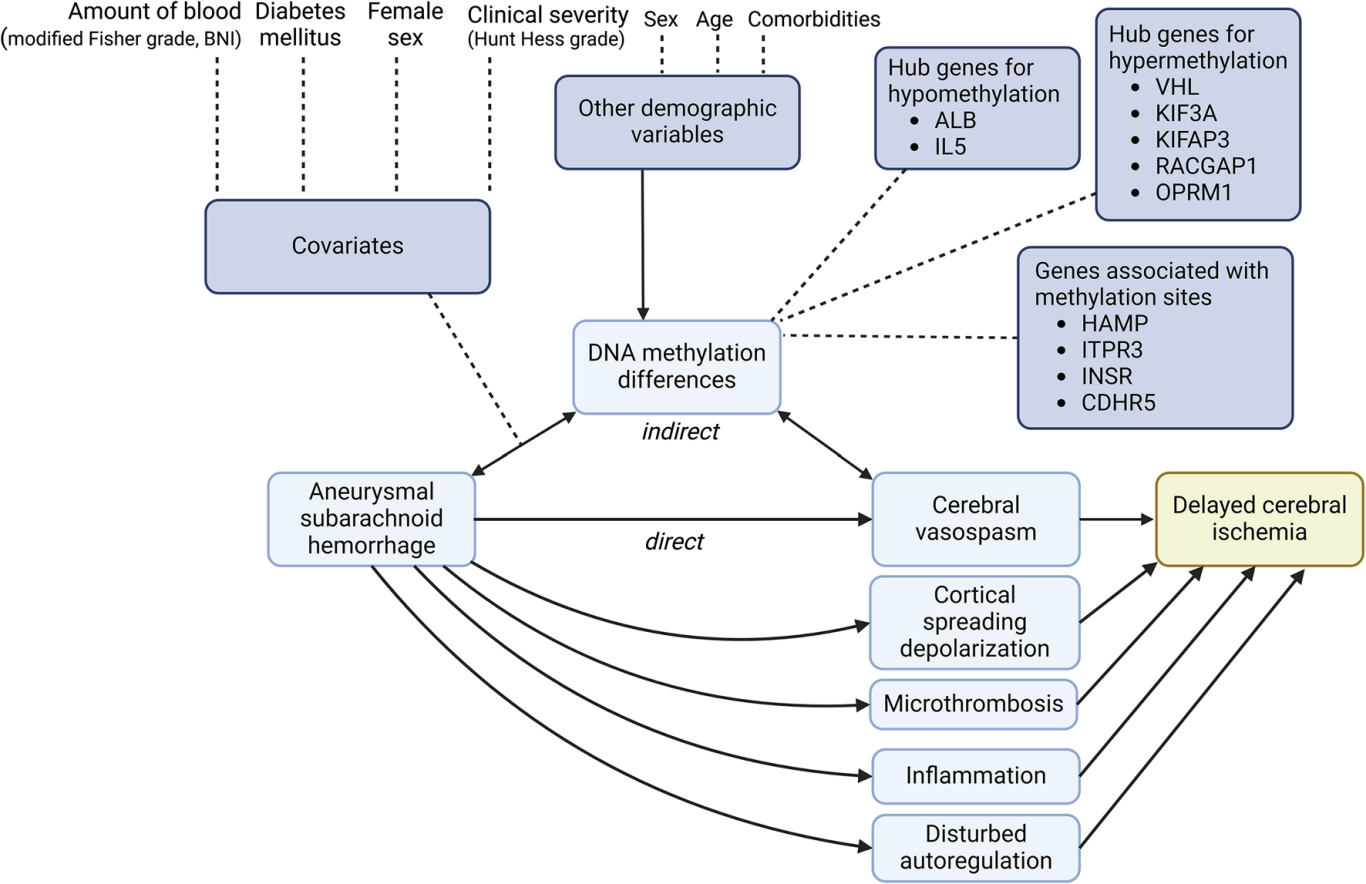
Direct acyclic graph (DAG) for aneurysmal subarachnoid hemorrhage, delayed cerebral ischemia and DNA methylation alterations. BNI – Barrows Neurological Institute grading scale. DMC – differentially methylated CpGs. DMR – differentially methylated regions. Solid lines indicate a causal path. Dashed lines indicate covariates, demographic variables, and listed genes with differential DNA methylation. Created with BioRender.com

In the final search wave, 70 records were found by two authors across all three databases. After de-duplication process, 26 unique titles and abstracts were left. Those were screened for eligibility. 7 full-text papers were evaluated, all of which fulfilled the inclusion criteria [11–17]. Figure 1 presents a flow diagram along with reasons for exclusion. Quality evaluation indicated that four papers were of high quality whereas three studies were of very high quality with the sample size estimation and sensitivity analysis being addressed the least frequently (see Supplementary Material 3). Five studies used a candidate gene approach [11–14, 16], three were epigenome-wide association studies (EWAS) [14, 16, 17], one utilized bioinformatics of the previous EWAS [15], with two studies using more than one approach [14, 16]. One research article evaluated epigenetic age (DNA-methylation biological age calculated as age acceleration) [17]. A tissue sample for DNA methylation profiling was whole blood in five studies [13–17] and cerebrospinal fluid in two [11, 12]. Table 1 summarizes study characteristics. As required by eligibility criteria, a comparison of DCI versus non-DCI was done in all articles. Methods of DNA extraction, DNA concentration and quality assessment, conversion, and methylation measurement are delineated in Supplementary Material 1. DNA input varied between 500 to 2000 ng. None of the sites overlapped. Bioinformatics analysis utilizing functional enrichment analysis, protein–protein interaction network, and module analysis of the data from the previously conducted EWAS identified hub genes for hypermethylation (VHL, KIF3A, KIFAP3, RACGAP1, OPRM1) and hypomethylation (ALB, IL5) in DCI after aSAH. Figure 2 presents direct acyclic graph for aneurysmal subarachnoid hemorrhage, delayed cerebral ischemia and DNA methylation alterations. Biological age acceleration analysis though epigenetic clocks did not detect differences in DCI. However, multivariate analysis indicated that subjects with *radiological* vasospasm had a lower age acceleration than patients without radiological vasospasm, finding significant differences in both Horvath’s and Levine’s clocks independently of the main confounding factors (Hunt Hess grade, sex, smoking, hypertension, diabetes mellitus and time since onset of symptoms). See Table 2 for the summary of key findings. As none of the differentially methylated CpGs overlapped across the studies, meta-analysis was not applicable.

**Table 1:** Basic characteristics of the eligible studies. aSAH – aneurysmal subarachnoid hemorrhage. CpG—cytosine-guanine dinucleotide. CSF – cerebrospinal fluid. EWAS – epigenome-wide association study. kb – kilobase. WFNS – World Federation of Neurological Surgeons grading scale

Study	Ethnicity	Approach	Targeted region	Epigenetic mechanism	Tissue	DCI definition	Time from aSAH to sample collection	Groups of comparison	No. of subjects	Subjects with DCI, *n* (%)	Females, *n* (%)	Mean age (SD)	Clinical severity, *n* (%)	Fisher grade, *n* (%)
Kim 2019	Asian	Candidate gene approach	Intergenic region upstream of ITPR3 gene with 1 CpG	DNA methylation	Whole blood	New neurologic deficit (motor weakness, sensory change, dysphasia, decreased consciousness) with CVS	Not specified	DCI vs non-DCI	42	14 (33,3%)	30 (71,4%)	58,07 (9,04)	Hunt Hess Grade 1–3: 25 (59,5%) Grade 4–5: 17 (40,5%)	Grade 1–2: 9 (21,4%) Grade 3–4: 33 (78,6%)
Heinsberg 2020	86,5% Caucasian	Candidate gene approach	Within the HAMP transcript region ± 2000 kb upstream and downstream (chr19:35,771,410:35,778,045) with 8 CpGs	DNA methylation	CSF	Co-occurrence of neurological deterioration (new and persistent > 1 h neurological deficit) and abnormal cerebral blood flow measured using cerebral angiogram or transcranial Doppler	Up to five time points over 14 days following aSAH	DCI vs non-DCI	249	116 (46,6%)	179 (71,89%)	53.4 (11,4)	WFNS Grade 1–3: 188 (72,3%) Grade 4–5: 72 (27,7%)	Grade 1–2: 78 (30%) Grade 3–4: 182 (70%)
Kim 2020	Asian	EWAS (discovery) Candidate gene approach (replication)	Epigenome-wide (~ 450,000 CpGs)	DNA methylation	Whole blood	New neurologic deficits (motor weakness, sensory change, dysphasia, decreased consciousness) with CVS	Not specified	DCI vs non-DCI	Discovery: 40 Replication: 36	Discovery: 13 (32,5%) Replication: 12 (33,3%)	Discovery: 25 (62,5%) Replication: 25 (69,1%)	56.2 (10,0)	Hunt Hess Grade 1–3: 27 (67,5%) Grade 4–5: 13 (32,5%)	Grade 1–2: 8 (20%) Grade 3–4: 32 (80%)
Heinsberg 2021	Discovery: 86,5% Caucasian; Replication: 91% Caucasian	Candidate gene approach	Transcript region of 36 genes ± 2000 base pairs upstream and downstream	DNA methylation	CSF	Co-occurrence of neurological deterioration (new and persistent > 1 h neurological deficit) and abnormal cerebral blood flow measured using cerebral angiogram or transcranial Doppler	Five time points over 14 days following aSAH	DCI vs non-DCI	Discovery: 260 Replication: 100	Discovery: 127 (48,8%) Replication: DCI not analyzed	Discovery: 179 (68,8%) Replication: DCI not analyzed	53,1 (11,0)	Not specified	Grade 1–2: 119 (33%) Grade 3–4: 241 (67%)
Liu 2021	Discovery: 86% Caucasian Replication: 90% Caucasian	EWAS (discovery) Candidate gene approach (replication)	Epigenome-wide (~ 450,000 CpGs)	DNA methylation	Whole blood	Not specified	Within 2 days following aSAH	DCI vs non-DCI	Discovery EWAS: 68 Discovery MethylSeq: 58 Replication: 175	Discovery EWAS: 35 (51%) Discovery: 28 (48%) Replication: 52 (30%)	Discovery EWAS: 48 (71%) Discovery: 39 (67%) Replication: 129 (74%)	52.9 (11.1)	Not specified	Grade 1–2: 88 (37,8%) Grade 3–4: 145 (62,2%)
Kim 2022	Asian	Bioinformatics analysis (Functional enrichment analysis, protein–protein interaction network and module analysis) of the previous EWAS	Epigenome-wide (~ 450,000 CpGs)	DNA methylation	Whole blood	New neurologic deficit (motor weakness, sensory change, dysphasia, or decreased consciousness or any changes greater than 2 points in the Glasgow Coma Scale or National Institutes of Health stroke scale); and the presence of severe angiographic vasospasm, defined as arterial narrowing over 50%	Not specified	DCI vs non-DCI	40 (same cohort as in Kim 2020)	13 (32,5%)	Discovery: 25 (62,5%) Replication: 25 (69,1%)	56.2 (10,0)	Hunt Hess Grade 1–3: 27 (67,5%) Grade 4–5: 13 (32,5%)	Grade 1–2: 8 (20%) Grade 3–4: 32 (80%)
Macias-Gómez 2024	85,6% Caucasian; 10,8% Hispanic; 3,6% Other	EWAS	Epigenome-wide (865,918 CpGs)	DNA methylation, epigenetic clocks	Whole blood	A focal neurological impairment or a decrease of at least 2 points in the Glasgow Coma Scale, lasting for at least 1 h or the presence of a new ischemic lesion not apparent in the first neuroimaging after aneurysm occlusion and not attributable to other neurological or systemic causes	Upon admission	DCI vs non-DCI	277	70	165 (66,8%)	55,0 (13,3)	Hunt Hess Grade 1–3: 213 (76,8%) Grade 4–5: 64 (23,2%)	Modified Fisher Grade 0–2 Grade 3–4:

**Table 2:** Key findings of the eligible studies. Chr – chromosome. DCI – delayed cerebral ischemia. EWAS – epigenome-wide association study. GOS – Glasgow Outcome Scale. N/A – Not applicable

Study	Sites tested	Sites suggestive or significant for DCI	Associated genes	Chr	Associated region of gene	Protein	Relation to island	Conclusion related to DCI
Kim 2019	cg12770425	cg12770425	ITPR3	6	Intergenic region	IP3R3	North shelf	Hypermethylation of the distal intergenic region located upstream of ITPR3 might be associated with DCI development in subjects with aSAH
Heinsberg 2020	cg04668516 cg02131995 cg18149657 cg23677000 cg04085447 cg17907567 cg26283059 cg27273033	cg26283059	HAMP	19	5’UTR	Hepcidin	South shelf	Non-adjusted as well as adjusted for cell-type heterogeneity analysis indicated methylation trajectory of cg26283059 (related to HAMP gene) being suggestively associated with DCI, but the threshold after Bonferroni correction was not reached
Kim 2020	Discovery: EWAS Replication: Regions of INSR and CDHR5 genes	In both EWAS and replication phases: 1. Region of INSR gene (includes cg00441765) 2. Region of CDHR5 gene (includes cg11464053)	1. INSR 2. CDHR5	1. 19 2. 11	1. Body 2. Body	1. Insulin receptor 2. Cadherin-related family member 5	1. North shelf 2. South shore	Subjects with DCI had significantly hypermethylated CpG sites associated with two genes: INSR (cg00441765) and CDHR5 (cg11464053)
Heinsberg 2021	Discovery: 183 CpG sites in 33 genes Replication: cg25713625 (STEAP3) cg08866780 (APP) cg08553327 (TNF)	None for DCI The below mentioned CpGs were suggestive or significant for Glasgow Outcome Scale for aSAH in general at follow up: 1. cg25713625 2. cg08866780 3. cg08553327	1. STEAP3 2. APP 3. TNF	1. 2 2. 21 3. 6	1. 3'UTR 2. TSS1500 3. 1stExon	1. STEAP3 metalloreductase 2. Amyloid precursor protein 3. Tumor necrosis factor	1. South shore 2. Island 3. Open sea	No associations with DCI in discovery cohort; therefore, DCI was not included in the analysis of the replication cohort 1. On days 1–14 post-aSAH, methylation trajectory of cg25713625 was significantly associated with Glasgow Outcome Scale after aSAH, but not with DCI 2. On days 1–14 post-aSAH, methylation trajectory of cg08866780 was suggestively associated with **radiological** vasospasm and GOS at 3 months but not with DCI 3. On days 1–14 post-aSAH, methylation trajectory of cg08553327 was suggestively associated with Glasgow Outcome Scale, but not with DCI occurrence
Liu 2021	Discovery: EWAS Replication: cg18031596 cg09396217 cg27616227 cg02536838 chr8: 108,510,324 (cg16318522)	None for DCI Replication yielded top signal for: 1. cg18031596 2. cg09396217 3. cg27616227 4. chr8: 108,510,324 (cg16318522)	ANGPT1	8	1. TSS200 2. TSS200 3. TSS200 4. TSS200	Angiopoietin-1 (ANG-1)	1. Open sea 2. Open sea 3. Open sea 4. Open sea	EWAS did not yield any CpGs significantly associated with DCI. The top signal was found in cg18031596 (annotated to ANGPT1). Replication of a gene candidate was marked as a failure—all four CpGs of different methylation status had an opposite effect direction in the replication (negative) analysis compared to the discovery (positive) EWAS, marking a failure of replication
Kim 2022	Bioinformatics analysis (Functional enrichment analysis, protein–protein interaction network and module analysis) of the previous EWAS	N/A	Hub genes for hypermethylation: 1. VHL 2. KIF3A 3. KIFAP3 4. RACGAP1 5. OPRM1 Hub genes for hypomethylation: 6. ALB 7. IL5	1. 3 2. 5 3. 1 4. 12 5. 6 6. 4 7. 5	N/A	1. von Hippel-Lindau disease tumor suppressor 2. Kinesin-like protein KIF3A 3. Kinesin-associated protein 3 4. Rac GTPase-activating protein 1 5. Mu-type opioid receptor 6. Albumin 7. Interleukin-5	N/A	Hub genes for hypermethylation (VHL, KIF3A, KIFAP3, RACGAP1, OPRM1) and hypomethylation (ALB, IL5) could be useful biomarkers for the early and accurate DCI detection
Macias-Gómez 2024	EWAS Hannum’s clock: 71 CpG sites Horvath’s clock: 353 CpG sites Levine’s clock: 513 CpG sites	No significant differences in age acceleration were found for DCI	N/A	N/A	N/A	N/A	N/A	No significant differences for DCI. Multivariate analysis indicated that subjects with **radiological** vasospasm had a lower age acceleration than patients without radiological vasospasm, finding significant differences in both Horvath’s and Levine’s clocks independently of the main confounding factors (Hunt Hess grade, sex, smoking, hypertension, diabetes mellitus and time since onset of symptoms)

## Discussion

**Fig. 3:**
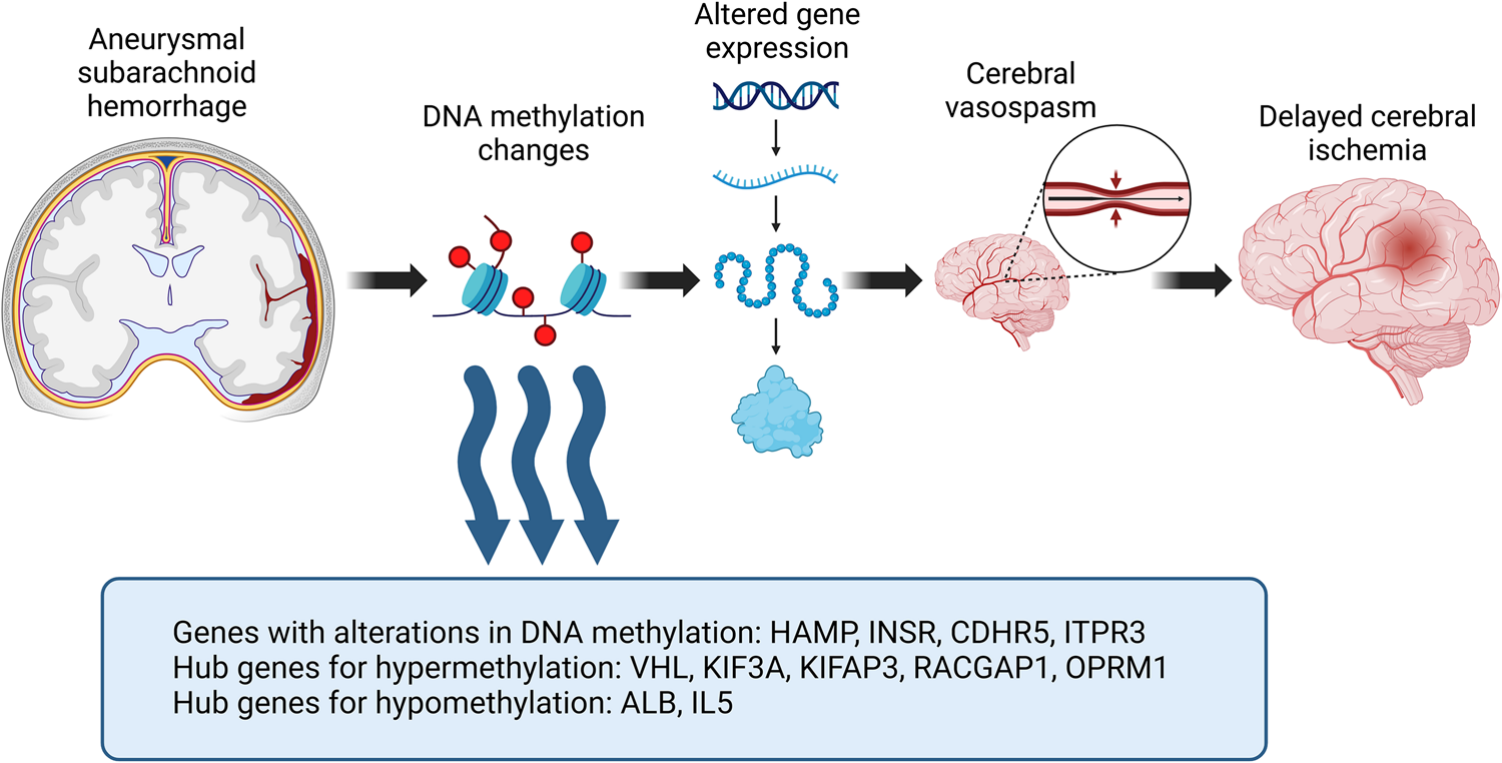
Conceptual diagram representing the epigenetic pathway from aneurysmal subarachnoid hemorrhage to delayed cerebral ischemia. Created with BioRender.com

This systematic review has identified four differentially methylated CpGs related to four different genes that could participate in development of CVS leading to delayed cerebral ischemia. Also, five hub genes for hypermethylation and two hub genes for hypomethylation were detected – see Fig. 3 for a conceptual diagram presenting epigenetic pathways for DCI after aSAH.

ITPR3 is a gene coding a protein inositol 1,4,5-triphosphate receptor type 3 – see Table 3. This protein serves as both a receptor for inositol triphosphate and calcium channel. The protein takes part in the pathway of endothelin receptor type B activation, which mediates vasodilation by enhancing activity of the eNOS. This biological function would elucidate its epigenetic involvement in cerebral vasospasm. One study [13] noticed significant hypermethylation of its distal intergenic region upstream involving cg12770425 in DCI group after aSAH vs non-DCI group: (median = 0.941; interquartile range [IQR] = 0.857–0.984) vs 0.670 [IQR = 0.543–0.761], respectively; *p* < 0.01). This was consistent with a reduced mRNA expression of ITPR3 in those subjects indicating dysfunction in the aforementioned pathway. Therefore, hypermethylation of cg12770425 decreases ITPR3 signaling and leads to inhibition of endothelin receptor activation and ultimately contributes to cerebral vasoconstriction.

**Table 3:** Genes related to differentially methylated sites in the context of delayed cerebral ischemia. aSAH – aneurysmal subarachnoid hemorrhage. BNI – Barrows Neurological Institute grading. CVS – cerebral vasospasm. DCI—delayed cerebral ischemia

Gene	Genomic location	Protein	Role and implications in cerebral vasospasm
HAMP	Chr19:35,280,716–35,285,143	Hepcidin	Significant role in iron homeostasis of the extravasated blood. Excessive iron in subarachnoid space not controlled by hepcidin is theorized to be one of the factors aggravating the injury after aSAH and leading to vasospasm. Explains high-grade subarachnoid hemorrhage (3–4 modified Fisher grade and BNI) being a risk factor for CVS and DCI
ITPR3	Chr6:33,620,365–33,696,574	Inositol 1,4,5-triphosphate receptor type 3	Takes part in the pathway of endothelin receptor type B activation, which mediates vasodilation by enhancing activity of the eNOS. Hypermethylation of cg12770425 decreases ITPR3 expression and leads to inhibition of this pathway followed by endothelin receptor inactivation and cerebral vasoconstriction
INSR	Chr19:7,112,255–7,294,414	Insulin receptor tyrosine kinase	INSR is present abundantly in cerebral cortex, striatum and olfactory bulbs. INSR signaling is involved in the regulation of cerebral glucose metabolism and neuronal function. Hypermethylation of cg00441765 leads to reduced INSR expression. aSAH patients who present with hyperglycemia or uncontrolled diabetes mellitus upon admission have increased inflammatory response. Hyperglycemia aggravates ischemic injury and is associated with DCI and poor clinical outcome in aSAH patients
CDHR5	Chr11:616,577–626,078	Cadherin-related family member 5	Intermicrovillar adhesion molecules forming heterophilic complexes on adjacent microvilli of the proximal renal tubules. Thus, they control packing of the microvilli in the epithelial cells. Hypermethylation of cg11464053 decreases CDHR5 expression and contributes to renal injury, disrupted fluid balance, and laboratory finding of hyponatremia. Hyponatremia in aSAH patients has been variably indicated as a risk factor for CVS, DCI or poor clinical outcome after aSAH

HAMP codes a protein hepcidin, which plays a significant role in iron homeostasis. It controls intestinal iron absorption as well as iron storage in macrophages. It was shown to have strong antimicrobial activity against Escherichia coli, Neisseria cinerea, and Staphylococcus aureus [18]. Extravasated haemoglobin is degraded into heme and globins. Heme is further catabolised into free iron, biliverdin, and carbon monoxide. Excessive iron in subarachnoid space not controlled by hepcidin is theorised to be one of the factors leading to vasospasm [11, 19]. Heinsberg et al. performed a unique study with group-based trajectory analysis of HAMP methylation pattern calculated across post-bleeding days 1 to 14 [11]. Analysis unadjusted for cell-type heterogeneity revealed non-significant (*p* > 0,05) association between DCI and distinct methylation trajectory of CpG sites cg18149657, whereas suggestive association was demonstrated for distinct methylation trajectory of cg26283059 (*p* = 0,01, but with Bonferroni correction the threshold was 0,002). Adjustment for cell-type heterogeneity presented similar results with methylation trajectory of cg26283059 being suggestively associated (*p* = 0,01) with DCI. For each CpG site, distinct methylation trajectories were not associated with *angiographic* vasospasm. Decreased HAMP signaling in massive subarachnoid hemorrhage and excessive iron accumulation would clarify pathophysiological basics on why high-grade Fisher (3 and 4) bleeding is associated with DCI [20].

CDHR5 is a novel gene coding cadherin-related family member 5 proteins that are intermicrovillar adhesion molecules forming heterophilic complexes on adjacent microvilli [21]. Thus, they control packing of the microvilli in the epithelial cells. One study evaluated epigenetic changes of CDHR5 in clinical vasospasm [14]. The gene was hypermethylated in subjects with DCI as compared to those without DCI (*p* = 0,017), which resulted in lower mRNA expression of CDHR5 in the DCI group. Hypermethylation of cg11464053 decreases CDHR5 expression and contributes to renal injury, disrupted fluid-electrolyte balance, and laboratory finding of hyponatremia. Hyponatremia in aSAH patients has been variably indicated as a risk factor for CVS, DCI, or poor clinical outcome after aSAH [22, 23].

INSR encodes an insulin receptor tyrosine kinase that takes part in pleiotropic actions of insulin and is expressed abundantly in cerebral cortex, striatum, and olfactory bulbs [24]. INSR signaling is involved in the regulation of cerebral glucose metabolism and neuronal function [25]. Hypermethylation of cg00441765 leads to reduced INSR expression. aSAH patients who present with hyperglycemia or uncontrolled diabetes mellitus upon admission have increased inflammatory response. Hyperglycemia aggravates ischemic injury and is associated with DCI and poor clinical outcome in aSAH patients [26]. Methylation status of INSR with relation to clinically evident vasospasm was estimated in one paper [14]. Hypermethylation of cg00441765 related to INSR was associated with DCI (*p* = 0,002). And likewise, mRNA expression of INSR was significantly reduced in the group of DCI as compared to the non-DCI (*p* < 0,001). There have been no studies so far on INSR methylation in specifically angiographic or ultrasonic vasospasm.

Seven hub genes were found to be associated with DCI: five hub genes for hypermethylation and two for hypomethylation. Hub genes are pivotal nodes within biological networks, particularly in gene regulation or protein–protein interaction networks. They may not be directly associated with vasoconstriction, yet their unique feature of a high degree of connectivity allows them to interact with many other genes. Functionally, the identified hub genes (VHL, KIF3A, KIFAP3, RACGAP1, OPRM1, ALB, IL5) are critical in maintaining the functional and structural integrity of the network. Alterations in hub genes can lead to significant alterations in the network's status, potentially resulting in diseases. They have potential clinical utility as a biomarker in early diagnosis or prognosis of DCI and may be targets for therapeutic intervention due to their influential role in gene regulation pathways. Similar utility has been studied in a variety of pathologies such as glioblastoma [27], sepsis [28], colorectal cancer [29], and others [30].

Epigenetic clocks are quantitative predictive models of biological age. They utilize measurement of DNA methylation at age-related CpG sites regressed against confounding factors to predict age acceleration residuals. A number of epigenetic clocks have been invented for this goal. The first epigenetic clock that was formulated is termed Hannum’s clock and it calculates DNA methylation of 71 CpG sites. Newer clocks such as Horvath’s or Levine’s take into account 353 and 513 CpG sites, respectively. The latter considers relevant clinical markers as well. In the study of Macias-Gomez et al., no significant differences in age acceleration were found regarding the presence of DCI. Multivariate analysis indicated that subjects with *radiological* vasospasm had significantly lower age acceleration than patients without *radiological* vasospasm in both Horvath’s and Levine’s clocks independently of the main confounding factors (Hunt Hess grade, sex, smoking, hypertension, diabetes mellitus and time since presentation of symptoms). Although no differences were found in age acceleration between DCI and non-DCI groups, a subgroup analysis of DCI patients indicated that those with both DCI and radiographic vasospasm had a biologically rejuvenated epigenetic profile (were less age-accelerated) than those with DCI but no radiographic vasospasm. This finding suggests two pathways for DCI – one involving CVS in the young, and the other without CVS in the elderly that might implicate other mechanisms of DCI such as cortical spreading depolarization, disturbed autoregulation, microthrombosis, or inflammation [31–34].

### Epigenetic modifications as therapeutic targets in DCI

Since CpG sites related to HAMP, INSR, CDHR5, and ITPR3 genes were found to be hypermethylated in DCI, epidrugs that inhibit DNA methyltransferases could potentially target those alterations. An example of such drugs is 5-aza-2′-deoxycytidine (2-deoxy-5-azaC; decitabine) which has been shown to reverse pathology in both cancer and non-cancerous diseases, for instance systemic lupus erythematosus or Sjogren’s syndrome [35, 36]. In DCI after aSAH, reaching normomethylation status of the relevant sites could lead to enhanced handling of iron from the extravasated blood by hepcidin, improved pathway signaling of endothelin receptor type B mediating vasodilation, or better glycemia and natremia homeostasis. In fact, inhibition of DNA methyltransferase (DNMT) or DNMT gene deletion was shown to be protective against delayed ischemic brain injury in mice [37]. The use of decitabine or azacytidine is limited by their poor chemical stability and relative toxicity [38]. This has been partly overcome by the introduction of zebularine, a DNMT inhibitor with a more stable structure and low cytotoxicity [38].

### Challenges and future perspectives

Epigenetics is a flourishing field with still little exploration in vascular neurosurgery. In classic understanding, DCI after aSAH was much related to clinical picture upon admission, bleeding severity, and comorbidities [39]. However, similarly to what was noted for the rupture of small intracranial aneurysms [40], radiomics might bring more accurate prediction, especially when combined with genomics and epigenomics into a powerful multi-omics analysis [41]. A similar approach could be applied to CVS and DCI to investigate multi-omics of this aSAH complication. Specifically, DNA methylation pattern could be supplemented with data from profiling of single-nucleotide polymorphisms (SNPs) in order to identify methylation quantitative trait loci (mQTLs), which have never been analysed in the context of CVS or DCI. mQTLs allow for examination of long-range interactions between relevant SNPs and CpG sites of hyper-/hypomethylation. In fact, mQTLs are the genetic variants that could influence such a methylation pattern. These SNP-methylation interactions might provide unique and novel data on the vasospasm mechanism. This, however, has not been addressed in the global literature of CVS after aSAH, yet. Similar crosstalk analysis was already done for entities such as ischemic stroke, coronary artery disease, schizophrenia, or Alzheimer’s disease [42–44]. To tackle this issue in the matter of vasospasm in aSAH, a nationally funded research project was started by the authors, entitled ‘Genetic and epigenetic fundamentals of cerebral vasospasm after aneurysmal subarachnoid hemorrhage’—grant number 2021/41/N/NZ2/00844.

### Limitations & generalizability

The first limitation refers to the scarcity of studies in the literature as only seven eligible papers have been published. Those seven studies were conducted by a total of four teams. In addition, definitions of DCI differed slightly across the studies, which might contribute to a lack of overlapping results. Notably, only one study included new ischemic lesions in neuroimaging in the definition. Unison in this matter is difficult as new imaging modalities are being suggested for DCI confirmation [45, 46]. Also, the review protocol was not published. Although protocol registration is recommended to avoid duplication, they are not routinely published as shown by the global survey [47]. Although mean age of the patients in all studies was similar, the generalizability of this review findings is limited by the fact that in four studies subjects with Caucasian race prevailed, whereas in the other three papers patients were of Asian descent. Such differences are of particular importance since ethnicity is a known factor influencing genetics and epigenetics in peripheral blood [48].

## Conclusions

This study has detected differential DNA methylation of CpG sites related to four genes, which associate with DCI after aSAH. Also, five hub genes for hypermethylation and two hub genes for hypomethylation were recognized. The identified methylation sites might potentially serve as a biomarker for early diagnosis of DCI after aSAH in future. However, a lack of overlapping results prompts the need for large-scale multicenter studies.

## Supplementary Information

Below is the link to the electronic supplementary material.

Supplementary file1 (XLSX 18 KB)

Supplementary file2 (DOCX 31 KB)

Supplementary file3 (XLSX 13 KB)

## Data Availability

The data that support the findings of this study are provided as Supplementary Materials.
